# 2-Chloro-*N*-methyl-*N*-[2-(methyl­amino)­phen­yl]acetamide

**DOI:** 10.1107/S1600536813000494

**Published:** 2013-01-12

**Authors:** Yu-Bin Bai, Xia-Hui Chen, Ya-Tuan Ma, An-Ling Zhang, Jin-Ming Gao

**Affiliations:** a College of Science, Northwest A&F University, Yangling Shaanxi 712100, People’s Republic of China

## Abstract

The title compound, C_10_H_13_ClN_2_O, was obtained as a by-product in the reaction of 2-chloro­methyl-1*H*-benzimidazole, dimethyl sulfate and toluene to synthesise 2-chloro­methyl-1-methyl­benzimidazole. The dihedral angle between the benzene ring and the acetamide group is 89.72  (6)° while that between the aromatic ring and the chloracetyl group is 84.40 (4)°. In the crystal, adjacent mol­ecules are linked by pairs of N—H⋯O hydrogen bonds into inversion dimers.

## Related literature

For the synthesis of similar compounds, see: Turner & Wood (1965 ▶); Bai *et al.* (2008 ▶).
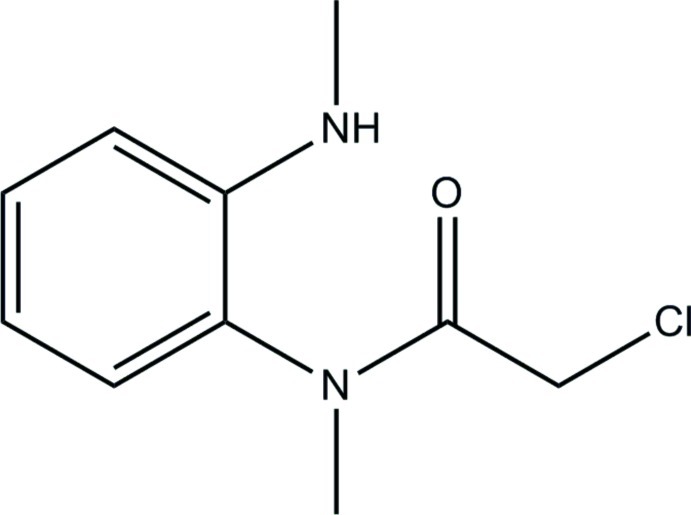


## Experimental

### 

#### Crystal data

C_10_H_13_ClN_2_O
*M* *_r_* = 212.67
Monoclinic, 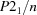

*a* = 9.2483 (18) Å
*b* = 6.6630 (13) Å
*c* = 17.622 (3) Å
β = 94.377 (2)°
*V* = 1082.8 (4) Å^3^
*Z* = 4
Mo *K*α radiation
μ = 0.32 mm^−1^
*T* = 296 K
0.50 × 0.35 × 0.21 mm

#### Data collection

Bruker APEXII CCD area-detector diffractometer
Absorption correction: multi-scan (*SADABS*; Sheldrick, 1996 ▶) *T*_min_ = 0.855, *T*_max_ = 0.935
7714 measured reflections
2011 independent reflections
1487 reflections with *I* > 2σ(*I*)
*R*_int_ = 0.023

#### Refinement

*R*[*F* ^2^ > 2σ(*F* ^2^)] = 0.045
*wR*(*F* ^2^) = 0.140
*S* = 1.01
2011 reflections
129 parameters
H-atom parameters constrained
Δρ_max_ = 0.29 e Å^−3^
Δρ_min_ = −0.24 e Å^−3^

### 

Data collection: *APEX2* (Bruker, 2004 ▶); cell refinement: *SAINT* (Bruker, 2004 ▶); data reduction: *SAINT*; program(s) used to solve structure: *SHELXS97* (Sheldrick, 2008 ▶); program(s) used to refine structure: *SHELXL97* (Sheldrick, 2008 ▶); molecular graphics: *SHELXTL* (Sheldrick, 2008 ▶); software used to prepare material for publication: *SHELXTL*.

## Supplementary Material

Crystal structure: contains datablock(s) I, global. DOI: 10.1107/S1600536813000494/nc2302sup1.cif

Structure factors: contains datablock(s) I. DOI: 10.1107/S1600536813000494/nc2302Isup2.hkl

Supplementary material file. DOI: 10.1107/S1600536813000494/nc2302Isup4.cdx

Supplementary material file. DOI: 10.1107/S1600536813000494/nc2302Isup4.cml

Additional supplementary materials:  crystallographic information; 3D view; checkCIF report

## Figures and Tables

**Table 1:** Hydrogen-bond geometry (Å, °)

*D*—H⋯*A*	*D*—H	H⋯*A*	*D*⋯*A*	*D*—H⋯*A*
N1—H1⋯O1^i^	0.86	2.23	2.926 (2)	138

Symmetry code: (i) 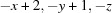.

**Fig. 1.**
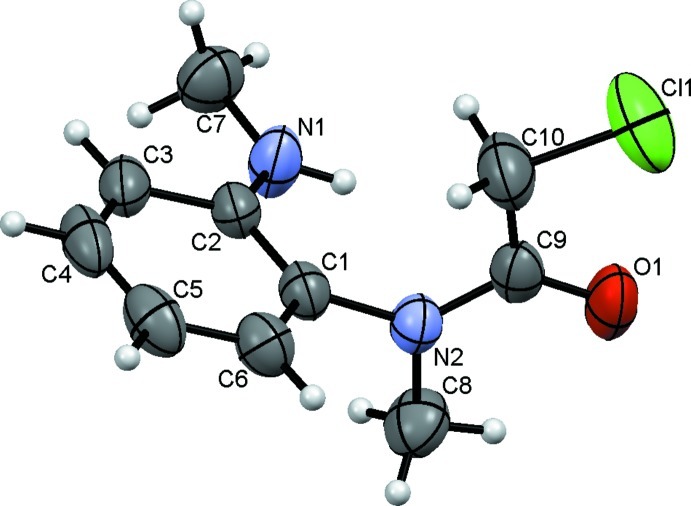
The molecular structure of the title compound with labeling and isplacement ellipsoids drawn at the 30% probability level.

## supplementary crystallographic information

### Experimental

2-chloromethyl-1*H*-benzimidazole (1.01 g, 6.07 mmol), toluene (10 ml),dimethyl sulfate (0.63 ml, 6.67 mmol) were refluxed for 3 h and the reaction was followed by TLC monitoring). After cooling 10 mL of water and an excess of ammonia were added. After filtration, the solution was extracted with chloroform (3 *x* 20 ml). The organic layer was dried over anhydrous sodium sulfate, concentrated and purified by columnchromatography on silica gel eluting with 4:1–3:1 petroleum ether-acetone. Crystals of the title compound were grown by slow evaporation of the solvent.

### Refinement

All H atoms were positioned with idealized geometry and refined isotropic with *U*_iso_(H) = 1.2 *U*_eq_(C,N) using a riding model.

### Crystal data

**Table:**

C_10_H_13_ClN_2_O	*F*(000) = 448
*M**_r_* = 212.67	*D*_x_ = 1.305 Mg m^−^^3^
Monoclinic, *P*2_1_/*n*	Mo *K*α radiation, λ = 0.71073 Å
*a* = 9.2483 (18) Å	Cell parameters from 2090 reflections
*b* = 6.6630 (13) Å	θ = 2.6–25.0°
*c* = 17.622 (3) Å	µ = 0.32 mm^−^^1^
β = 94.377 (2)°	*T* = 296 K
*V* = 1082.8 (4) Å^3^	Block, colourless
*Z* = 4	0.50 × 0.35 × 0.21 mm

### Data collection

**Table:**

Bruker APEXII CCD area-detector diffractometer	2011 independent reflections
Radiation source: fine-focus sealed tube	1487 reflections with *I* > 2σ(*I*)
Graphite monochromator	*R*_int_ = 0.023
phi and ω scans	θ_max_ = 25.5°, θ_min_ = 2.3°
Absorption correction: multi-scan (*SADABS*; Sheldrick, 1996)	*h* = −11→11
*T*_min_ = 0.855, *T*_max_ = 0.935	*k* = −8→8
7714 measured reflections	*l* = −21→21

### Refinement

**Table:**

Refinement on *F*^2^	Primary atom site location: structure-invariant direct methods
Least-squares matrix: full	Secondary atom site location: difference Fourier map
*R*[*F*^2^ > 2σ(*F*^2^)] = 0.045	Hydrogen site location: inferred from neighbouring sites
*wR*(*F*^2^) = 0.140	H-atom parameters constrained
*S* = 1.01	*w* = 1/[σ^2^(*F*_o_^2^) + (0.0697*P*)^2^ + 0.4335*P*] where *P* = (*F*_o_^2^ + 2*F*_c_^2^)/3
2011 reflections	(Δ/σ)_max_ < 0.001
129 parameters	Δρ_max_ = 0.29 e Å^−^^3^
0 restraints	Δρ_min_ = −0.24 e Å^−^^3^

### Special details

**Table:**

Geometry. All e.s.d.'s (except the e.s.d. in the dihedral angle between two l.s. planes)are estimated using the full covariance matrix. The cell e.s.d.'s are takeninto account individually in the estimation of e.s.d.'s in distances, anglesand torsion angles; correlations between e.s.d.'s in cell parameters are onlyused when they are defined by crystal symmetry. An approximate (isotropic)treatment of cell e.s.d.'s is used for estimating e.s.d.'s involving l.s. planes.
Refinement. Refinement of *F*^2^ against ALL reflections. The weighted *R*-factor *wR* andgoodness of fit *S* are based on *F*^2^, conventional *R*-factors *R* are basedon *F*, with *F* set to zero for negative *F*^2^. The threshold expression of*F*^2^ > σ(*F*^2^) is used only for calculating *R*-factors(gt) *etc*. and isnot relevant to the choice of reflections for refinement. *R*-factors basedon *F*^2^ are statistically about twice as large as those based on *F*, and *R*-factors based on ALL data will be even larger.

### Fractional atomic coordinates and isotropic or equivalent isotropic displacement parameters (Å^2^)

**Table:**

	*x*	*y*	*z*	*U*_iso_*/*U*_eq_	
C1	1.0326 (2)	0.5640 (3)	0.20070 (11)	0.0443 (5)	
C2	0.9122 (2)	0.6930 (3)	0.19791 (11)	0.0443 (5)	
C3	0.8956 (3)	0.8109 (3)	0.26283 (13)	0.0526 (6)	
H3	0.8181	0.8998	0.2633	0.063*	
C4	0.9928 (3)	0.7961 (4)	0.32578 (13)	0.0594 (7)	
H4	0.9793	0.8755	0.3681	0.071*	
C5	1.1092 (3)	0.6675 (4)	0.32793 (13)	0.0594 (7)	
H5	1.1733	0.6584	0.3711	0.071*	
C6	1.1285 (2)	0.5519 (4)	0.26424 (12)	0.0526 (6)	
H6	1.2072	0.4650	0.2644	0.063*	
C7	0.6897 (3)	0.8227 (4)	0.12831 (16)	0.0648 (7)	
H7A	0.6262	0.7888	0.1670	0.097*	
H7B	0.6398	0.8034	0.0791	0.097*	
H7C	0.7188	0.9605	0.1340	0.097*	
C8	0.9806 (3)	0.2351 (4)	0.14078 (16)	0.0655 (7)	
H8A	0.9882	0.1661	0.0934	0.098*	
H8B	0.8802	0.2518	0.1497	0.098*	
H8C	1.0281	0.1580	0.1814	0.098*	
C9	1.1273 (2)	0.4748 (4)	0.07858 (11)	0.0487 (5)	
C10	1.1969 (3)	0.6811 (4)	0.08199 (16)	0.0740 (8)	
H10A	1.2469	0.7003	0.1319	0.089*	
H10B	1.1217	0.7823	0.0753	0.089*	
Cl1	1.31970 (9)	0.71410 (17)	0.01279 (4)	0.0981 (4)	
N1	0.8153 (2)	0.6964 (3)	0.13566 (11)	0.0612 (6)	
H1	0.8305	0.6177	0.0984	0.073*	
N2	1.04930 (19)	0.4308 (3)	0.13748 (9)	0.0453 (4)	
O1	1.14232 (19)	0.3589 (3)	0.02587 (9)	0.0643 (5)	

### Atomic displacement parameters (Å^2^)

**Table:**

	*U*^11^	*U*^22^	*U*^33^	*U*^12^	*U*^13^	*U*^23^
C1	0.0489 (11)	0.0492 (12)	0.0357 (10)	−0.0029 (10)	0.0084 (9)	−0.0016 (9)
C2	0.0519 (12)	0.0455 (12)	0.0369 (11)	0.0002 (9)	0.0128 (9)	−0.0001 (9)
C3	0.0641 (14)	0.0470 (13)	0.0496 (13)	−0.0030 (10)	0.0228 (11)	−0.0044 (10)
C4	0.0806 (17)	0.0599 (15)	0.0400 (12)	−0.0238 (13)	0.0205 (12)	−0.0133 (11)
C5	0.0654 (15)	0.0733 (17)	0.0390 (12)	−0.0196 (13)	0.0006 (10)	−0.0026 (11)
C6	0.0528 (13)	0.0604 (14)	0.0444 (12)	−0.0039 (11)	0.0028 (10)	0.0001 (11)
C7	0.0593 (15)	0.0652 (16)	0.0700 (16)	0.0143 (12)	0.0053 (12)	−0.0009 (13)
C8	0.0724 (17)	0.0552 (15)	0.0696 (17)	−0.0062 (12)	0.0108 (13)	−0.0129 (12)
C9	0.0470 (12)	0.0606 (14)	0.0380 (11)	0.0130 (10)	0.0011 (9)	−0.0007 (10)
C10	0.0861 (19)	0.0806 (19)	0.0593 (16)	−0.0093 (15)	0.0314 (14)	−0.0046 (13)
Cl1	0.0836 (6)	0.1459 (9)	0.0684 (5)	−0.0247 (5)	0.0304 (4)	0.0038 (5)
N1	0.0624 (12)	0.0763 (14)	0.0449 (11)	0.0249 (10)	0.0041 (9)	−0.0101 (10)
N2	0.0484 (10)	0.0480 (10)	0.0398 (9)	0.0030 (8)	0.0054 (7)	−0.0055 (8)
O1	0.0742 (11)	0.0775 (12)	0.0417 (9)	0.0169 (9)	0.0064 (8)	−0.0138 (8)

### Geometric parameters (Å, º)

**Table:**

C1—C6	1.377 (3)	C7—H7B	0.9600
C1—C2	1.405 (3)	C7—H7C	0.9600
C1—N2	1.442 (3)	C8—N2	1.454 (3)
C2—N1	1.363 (3)	C8—H8A	0.9600
C2—C3	1.406 (3)	C8—H8B	0.9600
C3—C4	1.377 (4)	C8—H8C	0.9600
C3—H3	0.9300	C9—O1	1.224 (3)
C4—C5	1.374 (4)	C9—N2	1.341 (3)
C4—H4	0.9300	C9—C10	1.517 (4)
C5—C6	1.384 (3)	C10—Cl1	1.742 (3)
C5—H5	0.9300	C10—H10A	0.9700
C6—H6	0.9300	C10—H10B	0.9700
C7—N1	1.432 (3)	N1—H1	0.8600
C7—H7A	0.9600		
			
C6—C1—C2	121.60 (19)	H7B—C7—H7C	109.5
C6—C1—N2	119.48 (19)	N2—C8—H8A	109.5
C2—C1—N2	118.76 (18)	N2—C8—H8B	109.5
N1—C2—C3	122.7 (2)	H8A—C8—H8B	109.5
N1—C2—C1	120.60 (19)	N2—C8—H8C	109.5
C3—C2—C1	116.7 (2)	H8A—C8—H8C	109.5
C4—C3—C2	120.7 (2)	H8B—C8—H8C	109.5
C4—C3—H3	119.6	O1—C9—N2	123.3 (2)
C2—C3—H3	119.6	O1—C9—C10	122.0 (2)
C5—C4—C3	121.9 (2)	N2—C9—C10	114.78 (19)
C5—C4—H4	119.0	C9—C10—Cl1	112.58 (19)
C3—C4—H4	119.0	C9—C10—H10A	109.1
C4—C5—C6	118.3 (2)	Cl1—C10—H10A	109.1
C4—C5—H5	120.8	C9—C10—H10B	109.1
C6—C5—H5	120.8	Cl1—C10—H10B	109.1
C1—C6—C5	120.8 (2)	H10A—C10—H10B	107.8
C1—C6—H6	119.6	C2—N1—C7	124.2 (2)
C5—C6—H6	119.6	C2—N1—H1	117.9
N1—C7—H7A	109.5	C7—N1—H1	117.9
N1—C7—H7B	109.5	C9—N2—C1	124.03 (19)
H7A—C7—H7B	109.5	C9—N2—C8	119.30 (19)
N1—C7—H7C	109.5	C1—N2—C8	116.63 (18)
H7A—C7—H7C	109.5		
			
C6—C1—C2—N1	−177.2 (2)	N2—C9—C10—Cl1	170.27 (17)
N2—C1—C2—N1	−1.9 (3)	C3—C2—N1—C7	1.9 (4)
C6—C1—C2—C3	0.8 (3)	C1—C2—N1—C7	179.8 (2)
N2—C1—C2—C3	176.12 (18)	O1—C9—N2—C1	178.84 (19)
N1—C2—C3—C4	177.1 (2)	C10—C9—N2—C1	−1.0 (3)
C1—C2—C3—C4	−0.8 (3)	O1—C9—N2—C8	1.3 (3)
C2—C3—C4—C5	0.2 (3)	C10—C9—N2—C8	−178.6 (2)
C3—C4—C5—C6	0.6 (4)	C6—C1—N2—C9	−91.2 (3)
C2—C1—C6—C5	0.0 (3)	C2—C1—N2—C9	93.3 (3)
N2—C1—C6—C5	−175.32 (19)	C6—C1—N2—C8	86.4 (3)
C4—C5—C6—C1	−0.7 (3)	C2—C1—N2—C8	−89.1 (2)
O1—C9—C10—Cl1	−9.6 (3)		

### Hydrogen-bond geometry (Å, º)

**Table:**

*D*—H···*A*	*D*—H	H···*A*	*D*···*A*	*D*—H···*A*
N1—H1···O1^i^	0.86	2.23	2.926 (2)	138

Symmetry code: (i) −*x*+2, −*y*+1, −*z*.
